# A generalisation of the method of regression calibration and comparison with the Bayesian 2-dimensional Monte Carlo method

**DOI:** 10.21203/rs.3.rs-3700052/v1

**Published:** 2023-12-05

**Authors:** Mark P Little, Nobuyuki Hamada, Lydia B Zablotska

**Affiliations:** a Radiation Epidemiology Branch, National Cancer Institute, Bethesda, MD 20892-9778 USA; b Faculty of Health and Life Sciences, Oxford Brookes University, Headington Campus, Oxford, OX3 0BP, UK; c Biology and Environmental Chemistry Division, Sustainable System Research Laboratory, Central Research Institute of Electric Power Industry (CRIEPI), 1646 Abiko, Chiba 270-1194, Japan; d Department of Epidemiology and Biostatistics, School of Medicine, University of California, San Francisco, 550 16^th^ Street, 2^nd^ floor, San Francisco, CA 94143, USA

## Abstract

For many cancer sites it is necessary to assess risks from low-dose exposures via extrapolation from groups exposed at moderate and high levels of dose. Measurement error can substantially alter the shape of this relationship and hence the derived population risk estimates. Even in studies with direct measurement of low-dose exposures measurement error could be substantial in relation to the size of the dose estimates and thereby distort population risk estimates. Recently, much attention has been devoted to the issue of shared errors, common in many datasets, and particularly important in occupational settings.

In this paper we test a Bayesian model averaging method, the so-called Bayesian two-dimensional Monte Carlo (2DMC) method, that has been fairly recently proposed against a very newly proposed modification of the regression calibration method, which is particularly suited to studies in which there is a substantial amount of shared error, and in which there may also be curvature in the true dose response. We also compared both methods against standard regression calibration and Monte Carlo maximum likelihood. The Bayesian 2DMC method performs poorly, with coverage probabilities both for the linear and quadratic dose coefficients that are under 5%, particularly when the magnitudes of classical and Berkson error are both moderate to large (20%–50%). The method also produces substantially biased (by a factor of 10) estimates of both the linear and quadratic coefficients, with the linear coefficient overestimated and the quadratic coefficient underestimated. By comparison the extended regression calibration method yields coverage probabilities that are too low when shared and unshared Berkson errors are both large (50%), although otherwise it performs well, and coverage is generally better than the Bayesian 2DMC and all other methods. The bias of the predicted relative risk at a variety of doses is generally smallest for extended regression calibration, and largest for the Bayesian 2DMC method (apart from unadjusted regression), with standard regression calibration and Monte Carlo maximum likelihood exhibiting bias in predicted relative risk generally somewhat intermediate between the other two methods.

## Introduction

Moderate and high doses of ionising radiation are well established causes of most types of cancer ^1,2^. There is emerging evidence, particularly for leukaemia and thyroid cancer, of risk at low dose (<100 mGy) low-linear energy transfer (LET) radiation ^3–6^. For most other cancer endpoints it is necessary to assess risks via extrapolation from groups exposed at moderate and high levels of dose. A number of recent reviews of low dose risk have been conducted^7–13^. Crucial to the resolution of this area of uncertainty is the modelling of the dose-response relationship and the importance of both systematic and random dosimetric errors for analyses of the dose response, in particular in the Japanese atomic bomb survivors, which are central to evaluations of population risks by a number of committees assessing radiation risk ^1,14^. The problem of allowing for measurement error in dose when estimating dose-response relationships has been the subject of much interest in epidemiology ^15–30^ and it is known that measurement error can alter substantially the shape of this relationship and hence the derived population risk estimates ^31^.

Dose measurement errors can arise in a number of different ways. Berkson errors commonly result when true values are randomly distributed around a measured dial setting, as for example on a radiotherapy (RT) machine, $d_{i}$, with error $U_{i}$, so that $D_{i}=d_{i}+U_{i}$, implying that the $d_{i},U_{i}$ are independent. Alternatively, when the measured “doses”, $d_{i}$ are distributed at random around the true “doses”, $D_{i}$, so that $d_{i}=D_{i}+U_{i}$ so that the $D_{i},U_{i}$ are independent, then the “classical” error model results. A crucial difference between these error models is that in the Berkson model the nominal dose and error are independent, but in the classical error model it is the true dose and the error that are independent. In the atomic bomb survivors, radiation doses are estimated by using estimates of the position of the survivors in each city, orientation with respect to the bomb and other shielding structures, e.g., buildings. In this case the estimated doses, $d_{i}$, are thought to be lognormally distributed around the true doses, $D_{i}$ (i.e. classical error model)^32^. This assumption underlies many of the attempts that have been made to model dose error in the Japanese atomic bomb survivor Life Span Study (LSS) data ^15–19,21–23,29^.

Regression calibration has been frequently used to correct for the effects of classical error, and entails replacing terms for true dose, $D_{i}$, in regression models by the condititonal expectation of true dose given the oberved dose $d_{i},\;E[D_{i}|d_{i}]$^31^. Regression calibration works well when dose errors are not substantial and when there is no curvature in the dose response ^31^. However, substantial bias can result when regression calibration is applied when dose errors are substantial, also when errors are non-differential ^31,33,34^.

A modification of the regression calibration method has been very recently proposed which is particularly suited to studies in which there is a substantial amount of shared error, and in which there may also be curvature in the true dose response ^35^. This so-called extended regression calibration (ERC) method can be used in settings where there is a mixture of Berkson and classical error ^35^. In fits to synthetic datasets in which there is substantial upward curvature in the true dose response, and varying (and sometimes substantial) amounts of classical and Berkson error, the ERC method generally outperformed both standard regression calibration, Monte Carlo maximum likelihood (MCML) and unadjusted regression, particularly with respect to the coverage probabilities of the quadratic coefficient, particularly for larger magnitudes of the Berkson error, whether this is shared or unshared ^35^.

A Bayesian model averaging (BMA) method has been recently proposed, the so-called 2 dimensional Monte Carlo with Bayesian model averaging (2DMC with BMA) method ^28^, which has been used in fits to radiation thyroid nodule data ^36^. In the present paper we shall assess the performance of the 2DMC with BMA method against other methods of correction for dose error using simulated data. The simulated data we shall use is exactly as per the previous report ^35^.

## Methods

### Synthetic data used for assessing corrections for dose error

The methods used closely parallel those of the previous paper ^35^. We used the publicly available version of the leukaemia and lymphoma data of Hsu *et al* ^37^ to guide construction of a synthetic dataset, which we provide in outline in Table 1. Specifically we used the person year distribution by bone marrow dose groups 0–0.07, 0.08–0.19, 0.20–0.99, 1.00–2.49, ≥2.50 Gy. The central estimates of dose we assumed are close to the person year weighted means of these groups, and as given in Table 1, although for the uppermost dose group we assigned a central estimate of 2 Gy. The numbers of persons are close to the scaled sum of person year in these dose groups, scaling by a factor of 0.002. We assumed a composite Berkson-classical error model in which the true dose $D_{\mathrm{true},i,j}$ and the surrogate dose $D_{\mathrm{surr},i,j}$ to individual i (in dose group $k_{i}$) in simulation j are given by:

(1) $$D_{\mathrm{true},i,j}=D_{\mathrm{cent},k_{i}}exp\left[-0.5\left(\sigma_{\mathrm{share,Berkson}}{\;}^{2}+\sigma_{\mathrm{unshare,}\mathrm{Berkson}}{\;}^{2}\right)\right]exp\left[\sigma_{\mathrm{share,Berkson}}\varepsilon_{j}+\sigma_{\mathrm{unshare},Berkson}\delta_{i,j}\right]$$

(2) $$D_{\mathrm{surr},ij}=D_{\mathrm{cent,}k_{i}}exp\left[-0.5\left(\sigma_{\mathrm{share,Class}}{\;}^{2}+\sigma_{\mathrm{unshare,Class}}{\;}^{2}\right)\right]exp\left[\sigma_{\mathrm{share,Class}}\mu_{j}+\sigma_{\mathrm{unshare,Class}}\kappa_{i,j}\right]$$

The variables $\varepsilon_{j},\delta_{i,j},\mu_{j},\kappa_{i,j}$ are independent identically distributed N(0,1) random variables. The factors $D_{\mathrm{cent},k_{i}},\;D_{\mathrm{cent},k_{i}}$ are the central estimates of dose, as given in Table 1. The factors $exp\left[-0.5\left(\sigma_{\mathrm{share},\mathrm{Berkson}}{\;}^{2}+\sigma_{\mathrm{unshare},\;\mathrm{Berkson}}{\;}^{2}\right)\right]$ and $exp\left[-0.5\left(\sigma_{\mathrm{share},\mathrm{Class}}{\;}^{2}+\sigma_{\mathrm{unshare},\mathrm{Class}}{\;}^{2}\right)\right]$ ensure that the distributions given by (1) and (2) have theoretical mean that coincides with the central estimates $D_{\mathrm{cent},k_{i}}$. This composite Berkson-classical error model is suggested by a similar (but purely additive) model proposed by Reeves *et al* ^20^, whereas the errors in our model are of multiplicative form; the model of course ensures that the simulated doses are always positive. The model has the feature that when the Berkson error geometric standard deviations (GSD) are set to 0 $\left(\sigma_{\mathrm{share},\mathrm{Berkson}}=\sigma_{\mathrm{unshare},\mathrm{Berkson}}=0\right)$ the model reduces to one with classical error (a mixture of shared and unshared); likewise when the classical error GSDs are set to 0 $\left(\sigma_{\mathrm{share},\mathrm{Class}}=\sigma_{\mathrm{unshare},\mathrm{Class}}=0\right)$ the model reduces to one with pure Berkson error (a mixture of shared and unshared).

We generated a number of different versions of the dose data, with GSD $\sigma_{\mathrm{share},\mathrm{Berkson}},\;\sigma_{\mathrm{unshare},\mathrm{Berkson}},\;\sigma_{\mathrm{share},\mathrm{Class}},\;\sigma_{\mathrm{unshare},\mathrm{Class}}$ taking values of 0.2 (20%) or 0.5 (50%). This individual dose data was then used to simulate the distribution of N=250 cancers for each of m=1000 simulated datasets, indexed by j, using a model in which the assumed probability of being a case for individual i is given by:

(3) $$exp\left[\kappa_{j}\right]\left[1+\alpha D_{\mathrm{true},i,j}+\beta D_{\mathrm{true},i,j}{\;}^{2}\right]$$

the scaling constant $\kappa_{j}$ being chosen for each simulation (but not for the Bayesian model fits) to make these sum to 1. We assumed coefficients $\alpha =0.25/Gy,\;\beta =2/{Gy}^{2}$, close to the values derived from fits of a similar model to the 237 leukaemias in the data of Hsu *et al* ^37^.

A total of m=1000 samples were taken of each type of dose, as given by expressions (1) and (2). A total of n=500 simulations of these dose+cancer ensembles were used to fit models and evaluate fitted model means and coverage probability. Having derived synthetic individual level data, for the purposes of model fitting, for all models except MCML and 2DMC with BMA, the data were then collapsed (summing cases, averaging doses) into the 5 dose groups given in Table 1. Poisson linear relative risk generalised linear models ^38^ were fitted to this grouped data, with rates given by expression (3), using as offsets the number per group in Table 1. Models were fitted using five separate methods:

1. unadjusted – using only the mean surrogate doses per group given by group means of the samples generated by expression (2), using a single sampled dose per individual for each of m=500 dose+cancer ensembles;
2. regression calibration adjusted – using the mean true doses per group given by group means of the samples generated by expression (1), averaged over the n=1000 dose samples, for each of m=500 dose+cancer ensembles;
3. ERC adjusted – using the mean true doses per group given by group means of the samples generated by expression (1), averaged over the n=1000 dose samples, for each of m=500 dose+cancer ensembles, and with additional adjustments to the likelihood outlined in Appendix A;
4. MCML, using the full set of mean true doses per group, the mean doses per group for each simulation being given by group means of the samples generated by expression (1), averaged over the n=1000 dose samples.
5. 2DMC with BMA, using the full set of mean doses as for MCML and fitted using Bayesian Markov Chain Monte Carlo (MCMC) methods.

For the Bayesian model (5), associated with the dose vector is a vector of probabilities $p_{j},\;j=1,\ldots ,1000$ which is generated using variables $\lambda_{j},\;j=1,\ldots ,999$, so that:

(4) $$p_{j}=\frac{\exp\left[\lambda_{j}\right]}{1+\sum_{k=1}^{999}\exp\left[\lambda_{k}\right]},j=1,\ldots ,999,\;p_{1000}=\frac{1}{1+\sum_{k=1}^{999}\exp\left[\lambda_{k}\right]}$$

All main model parameters $\left(\alpha ,\beta ,\kappa ,\lambda_{k}\right)$ had normal priors, with mean 0 and standard deviation (SD) 1000. The Metropolis-Hastings algorithm was used to generate samples from the posterior distribution. Random normal proposal distributions were assumed for all variables, with SD of 0.2 for κ and 1 for α,β, and SD 2 for all $\lambda_{k}$. The $\lambda_{k}$ were proposed in blocks of 10. Two separate chains were used, in order to compute the Brooks-Gelman-Rubin (BGR) convergence statistic ^39,40^. The first 1000 simulations were discarded, and a further 1000 simulations taken for sampling. The proposal SDs and number of burn-in sample were chosen to give mean BGR statistics (over the 500 simulated datasets) that were in all cases less than 1.03 and acceptance probabilities of about 30% for the main model parameters (α,β,κ), suggesting good mixture and likelihood of chain convergence. For models (1)–(4) confidence intervals were derived using the profile likelihood ^38^ and for model (5) Bayesian uncertainty intervals were derived. The Fortran 95–2003 program used to generate these datasets and perform Poisson and Bayesian MCMC model fitting, and the relevant steering files employed to control this program are given in online Appendix B. Using the mean coefficients for each model and error scenario over the 500 simulated datasets, $\left(\alpha_{\mathrm{mean}},\beta_{\mathrm{mean}}\right)$, the percentage mean bias in predicted relative risk (RR) is calculated, via:

(5) $$100\left[\left(1+\alpha_{\mathrm{mean}}D_{\mathrm{pred}}+\beta_{\mathrm{mean}}D_{\mathrm{pred}}{\;}^{2}\right)/\left(1+\alpha D_{\mathrm{pred}}+\beta D_{\mathrm{pred}}{\;}^{2}\right)-1\right]$$

This was evaluated for two values of predicted dose, $D_{\mathrm{pred}}=0.1\;Gy$ and $D_{\mathrm{pred}}=1\;Gy$.

### Data availability statement

The datasets generated and analysed in the current study are available by running the Fortran 95/2003 program **fitter_shared_error_simulation_reg_cal_Bayes.for**, given in the online web repository, with any of the five steering input files given there. All are described in Appendix B.

The datasets are temporarily stored in computer memory, and the program uses them for fitting the Poisson models described in the Methods section.

## Results

As shown in Table 2, the coverage probabilities of all methods apart from 2DMC with BMA for the linear coefficient α are near the desired 95% level, irrespective of the magnitudes of assumed Berkson and classical error, whether shared or unshared. As would be expected for all methods save the uncorrected regression the magnitude of classical error makes no difference, since all regression correction methods use doses based on the true (rather than surrogate) dose as given by Eq. (1), without the classical error component. However, the coverage probabilities for the quadratic coefficient β are generally too low for the unadjusted and regression calibration methods, particularly for larger magnitudes of Berkson error (with GSD=50%), whether this is shared or unshared (Table 2). The ERC method also yields coverage probabilities that are somewhat too low when shared and unshared Berkson errors are both large (with GSD=50%), although otherwise it performs well, and coverage is uniformly better than all other methods (Table 2). In contrast MCML yields coverage probabilities for β that are uniformly too high (Table 2).

By contrast the coverage probabilities of both the linear coefficient α and the quadratic coefficient β for the 2DMC with BMA method are generally much too low, and when shared Berkson error is large (50%) the coverage probabilities do not exceed 5%.

Table 3 shows the coefficient mean values, averaged over all 500 simulations. A notable feature is that for larger values of Berkson error, the linear coefficient α for 2DMC with BMA is substantially overestimated, and the quadratic dose coefficient β substantially underestimated, both by factors of about 10. For all other methods apart from ERC the estimates of the quadratic coefficient β are upwardly biased, but not by such large amounts. There is upward bias in estimates of both α and β in the unadjusted analysis (using surrogate dose) even when there are no Berkson errors, for various magnitudes of classical errors, as shown by the first four rows of Table 3. As above, and as would be expected, for all models apart from the uncorrected one, the magnitude of classical error makes no difference.

Table 4 shows that the bias in RR for ERC evaluated either at 0.1 Gy or 1 Gy does not exceed 20%. Regression calibration performs somewhat worse, with bias ~40% when shared and unshared Berkson error are large (50%) for predictions at 1 Gy, although otherwise under 25%. For all but the smallest shared and unshared Berkson errors (both 0% or 20%) MCML has bias about ~25–45% at 1 Gy, although under 3% otherwise. 2DMC with BMA performs somewhat worse, with bias of ~30–45% when Berkson errors are large, whatever the evaluated dose. Unadjusted regression yields the most severe bias, which exceeds 50% in many cases for predictions at 1 Gy, and when shared or unshared classical errors are large (50%) often exceeds 100% (Table 4).

## Discussion

We have demonstrated that the 2DMC with BMA method performs poorly, with coverage probabilities both for the linear and quadratic dose coefficients that are under 5%, particularly when the magnitudes of classical and Berkson error are both moderate to large (20%–50%) (Table 2). This method also produces substantially biased (by a factor of 10) estimates of both the linear and quadratic coefficients, with the linear coefficient overestimated and the quadratic coefficient underestimated (Table 3). By comparison the ERC method yields coverage probabilities that are too low when shared and unshared Berkson errors are both large (50%), although otherwise it performs well, and coverage is generally better than for Bayesian 2DMC with BMA and all other methods (regression calibration, MCML, unadjusted regression). The upward bias in estimates of the α coefficient and the downward bias in the estimates of the β coefficient, at least for larger magnitudes of error (Table 3) largely explains the poor coverage in these cases (Table 2). The bias of the predicted RR at a variety of doses is generally smallest for ERC, and largest (apart from unadjusted regression) for 2DMC with BMA, with standard regression calibration and MCML exhibiting bias in predicted RR generally somewhat intermediate between the other two methods (Table 4).

The form of the 2DMC with BMA model that we fit differs slightly from that employed by Kwon *et al* ^28^ which uses an approximate Monte Carlo sampler, the so-called stochastic approximation Monte Carlo (SAMC) method of Liang *et al* ^41^. Unfortunately Kwon *et al* do not provide enough information to infer the precise form of SAMC that was used by them ^28^. It is possible that the SAMC implementation used by Kwon *et al* ^28^ may behave differently from the more standard implementation of BMA given here. Kwon *et al* ^28^ report results of a simulation study that tested the 2DMC with BMA method against what they term “conventional regression”, which may have been regression calibration. They did not assess performance against MCML. Kwon *et al* ^28^ report generally better performance of 2DMC with BMA against the regression calibration alternative.

The 2DMC with BMA method relies on Monte Carlo simulations from a Monte Carlo dosimetry system (MCDS). The key aspect is that ensembles of doses ${\left(D_{ijk}\right)}_{j=1k=1}^{N\;n_{j}}$ are produced for all individuals for a large number of scenarios i,1≤i≤M. However, unlike other uses of MCDS it is assumed that only one of the dose scenarios i, and therefore one of the sets of dose realisations ${\left(D_{ijk}\right)}_{j=1k=1}^{N\;n_{j}}$ is the correct one. Essentially this method therefore assumes something like a combination of functional and structural approaches – there are assumed to be random errors in the data, but certain parameters are assumed fixed (but unknown). The BMA approach is used to reweight the scenarios depending on the goodness of fit ^28^. So realisations where the risk-dose relationship was linear would be much more highly weighted than realisations where this was not the case. The contrast with MCML is quite pronounced - MCML works by averaging the likelihood in one go and then maximising the averaged likelihood with respect to the parameters of interest. The 2DMC with BMA method appears designed for applications where there is a substantial amount of shared error. This method has been applied to analysis of thyroid nodules in a dataset of persons exposed to atmospheric weapons tests in the former Soviet Union ^36^. The method has been much discussed ^42^. Stram *et al* ^42^ suggested that the method will produce substantially upwardly biased estimates of risk, also that the coverage may be poor, somewhat confirming our own findings; although we do not always find upward bias, the coverage of both regression coefficients is uniformly poor (Tables 2, 3). The implementation of the methodology presently relies on proprietary software, and has only been used by the group that developed it. Another substantial problem with the method is the use of BMA, reflecting general criticism made of this class of models in the literature ^43,44^. An implicit assumption of BMA is that one of the underlying models is the “true” one with convergence guaranteed to the “true” model ^45^.

As previously discussed^35^ the defects in the standard type of regression calibration that our modelling has revealed are not too surprising, as it is well known that this method can break down when dose error is substantial ^31^, as it is in many of our scenarios. Nevertheless the method has the considerable advantage that once the conditional expectations have been derived conventional statistical software can be used to perform regressions. The method has been successfully applied to the LSS cohort by a number of investigators ^15–19,46^ and has also been used in a few other radiation exposed groups ^25^. There have also been extensive applications in the non-radiation literature, reviewed by Carroll *et al* ^31^ and more recently in a series of papers by Shaw *et al* ^33,34^. Calibration approaches that take account of mixtures of Berkson and classical error have also been developed and used to fit domestic radon case-control data ^20^. More surprising is the relative weakness of MCML, which is discussed at greater length elsewhere^35^.

A Bayesian approach to the measurement error problem has been developed over the last 30 years which rests on the formulation of conditional independence relationships between different model components^47,48^, following the general structure outlined by Clayton ^49^. These are quite distinct form the 2DMC with BMA method. In the canonical Bayesian approach three basic sub-models are distinguished and linked: the disease model, the measurement model and the exposure model. The power of this Bayesian approach, as with MCML, is that the dosimetric uncertainty is (in principle) reflected in the variability of the model parameters relating dose to health effects. An adapted Bayesian method of correction for measurement error has been fitted to various versions of the LSS mortality data ^21,23,29^, also to an older version of the LSS incidence data ^22^. Derivation of the posterior distribution is generally analytically infeasible, and relies on the MCMC algorithm, specifically the Metropolis sampler, which converges to the posterior distribution of the risk parameters. However, the speed of convergence is not known, and in practice one relies on a number of *ad hoc* tests of convergence such as the Brooks-Gelman-Rubin statistic ^39,40^ and other less formal methods, e.g., inspection of caterpillar plots. As such all one can know is when convergence has not taken place. Flexible and efficient software exists to run this on a number of platforms e.g. OpenBUGS ^50^ or rjags ^51^. The method is exceptionally computationally burdensome. As with all Bayesian methods, in particular the 2DMC with BMA method used here, the choice of prior is critical.

### Conclusions

We have demonstrated that the 2DMC with BMA method performs poorly, with coverage probabilities both for the linear and quadratic dose coefficients that are under 5%, particularly when the magnitudes of classical and Berkson error are both moderate to large (Table 2). This method also produces substantially biased (by a factor of 10) estimates of both the linear and quadratic coefficients, with the linear coefficient overestimated and the quadratic coefficient underestimated (Table 3). By comparison the recently developed ERC method^35^ yields coverage probabilities that are too low when shared and unshared Berkson errors are both large (50%), although otherwise it performs well, and coverage is generally better than for Bayesian 2DMC with BMA and all other methods. The bias of the predicted RR at a variety of doses is generally smallest for ERC, and largest for 2DMC with BMA, with standard regression calibration and MCML exhibiting bias in predicted RR generally somewhat intermediate between the other two methods (Table 4).

## Figures and Tables

**Table 1.** Assumed distribution of persons by radiation dose group, based in part on distribution of person years in the Japanese atomic bomb survivor Life Span Study ^37^

Dose group	Central estimate of dose (Gy)	Scaled numbers of persons

1	0.01	2591
2	0.1	334
3	0.5	438
4	1.5	102
5	2	6

**Table 2.** Coverage probability of profile likelihood confidence intervals for fits of linear-quadratic model. Coverage probability evaluated using *m*=500 dose+cancer simulations. All but the rightmost two columns are taken from the paper of Little *et al*^35^.

Magnitude of error distribution (GSD)				Sample Pearson correlation coefficient between individual true doses	Unadjusted model		Regression calibration adjusted		Extended regression calibration adjusted		Monte Carlo maximum likelihood		Bayesian 2DMC with BMA	
					Coverage %		Coverage %		Coverage %		Coverage %		Coverage %	
Unshared Berkson error	Shared Berkson error	Unshared classical error	Shared classical error		*α*	*β*	*α*	*β*	*α*	*β*	*α*	*β*	*α*	*β*

0%	0%	20%	20%	NA	95.0	80.8	95.2	94.8	95.2	94.8	95.2	94.8	93.6	95.0
0%	0%	20%	50%	NA	94.4	55.4	95.2	94.8	95.2	94.8	95.2	94.8	93.6	95.0
0%	0%	50%	20%	NA	94.4	79.6	95.2	94.8	95.2	94.8	95.2	94.8	93.6	95.0
0%	0%	50%	50%	NA	94.6	55.0	95.2	94.8	95.2	94.8	95.2	94.8	93.6	95.0
20%	20%	20%	20%	0.50	95.0	80.0	95.4	94.0	94.8	98.4	95.4	99.0	92.4	94.2
20%	20%	20%	50%	0.50	94.8	53.6	95.4	94.0	94.8	98.4	95.4	99.0	92.4	94.2
20%	20%	50%	20%	0.50	94.6	78.2	95.4	94.0	94.8	98.4	95.4	99.0	92.4	94.2
20%	20%	50%	50%	0.50	94.6	52.2	95.4	94.0	94.8	98.4	95.4	99.0	92.4	94.2
20%	50%	20%	20%	0.84	93.6	77.4	94.4	85.6	95.4	94.8	95.0	100.0	4.6	1.6
20%	50%	20%	50%	0.84	93.2	48.4	94.4	85.6	95.4	94.8	95.0	100.0	4.6	1.6
20%	50%	50%	20%	0.84	93.8	75.4	94.4	85.6	95.4	94.8	95.0	100.0	4.6	1.6
20%	50%	50%	50%	0.84	93.8	48.2	94.4	85.6	95.4	94.8	95.0	100.0	4.6	1.6
50%	20%	20%	20%	0.15	94.2	76.6	94.0	86.0	94.4	94.8	95.0	99.2	88.0	96.8
50%	20%	20%	50%	0.15	93.4	48.4	94.0	86.0	94.4	94.8	95.0	99.2	88.0	96.8
50%	20%	50%	20%	0.15	94.0	75.6	94.0	86.0	94.4	94.8	95.0	99.2	88.0	96.8
50%	20%	50%	50%	0.15	94.0	49.0	94.0	86.0	94.4	94.8	95.0	99.2	88.0	96.8
50%	50%	20%	20%	0.45	95.4	64.0	95.4	67.8	95.0	80.4	96.4	100.0	0.8	3.8
50%	50%	20%	50%	0.45	95.0	40.0	95.4	67.8	95.0	80.4	96.4	100.0	0.8	3.8
50%	50%	50%	20%	0.45	94.4	64.6	95.4	67.8	95.0	80.4	96.4	100.0	0.8	3.8
50%	50%	50%	50%	0.45	94.2	40.0	95.4	67.8	95.0	80.4	96.4	100.0	0.8	3.8

Notes: GSD, geometric standard deviation.

**Table 3.** Mean over *m*=500 dose+cancer simulations of regression coefficients in fits of linear-quadratic model. All but the rightmost two columns are taken from the paper of Little *et al*^35^.

Magnitude of error distribution (GSD)				Unadjusted		Regression calibration		Extended regression calibration		Monte Carlo maximum likelihood		Bayesian 2DMC with BMA	
				ERR/Gy	ERR/Gy^2^	ERR/Gy	ERR/Gy^2^	ERR/Gy	ERR/Gy^2^	ERR/Gy	ERR/Gy^2^	ERR/Gy	ERR/Gy^2^
Unshared Berkson error	Shared Berkson error	Unshared classical error	Shared classical error	*α*	*β*	*α*	*β*	*α*	*B*	*α*	*β*	*α*	*β*

0%	0%	20%	20%	0.221	2.278	0.196	2.061	0.196	2.061	0.196	2.061	0.362	2.072
0%	0%	20%	50%	0.288	4.168	0.196	2.061	0.196	2.061	0.196	2.061	0.362	2.072
0%	0%	50%	20%	0.255	2.260	0.196	2.061	0.196	2.061	0.196	2.061	0.362	2.072
0%	0%	50%	50%	0.328	4.136	0.196	2.061	0.196	2.061	0.196	2.061	0.362	2.072
20%	20%	20%	20%	0.220	2.469	0.195	2.233	0.125	2.132	0.288	2.207	0.891	1.672
20%	20%	20%	50%	0.287	4.523	0.195	2.233	0.125	2.132	0.288	2.207	0.891	1.672
20%	20%	50%	20%	0.255	2.451	0.195	2.233	0.125	2.132	0.288	2.207	0.891	1.672
20%	20%	50%	50%	0.328	4.492	0.195	2.233	0.125	2.132	0.288	2.207	0.891	1.672
20%	50%	20%	20%	0.262	2.983	0.227	2.707	0.109	2.393	0.370	3.007	2.902	0.180
20%	50%	20%	50%	0.354	5.426	0.227	2.707	0.109	2.393	0.370	3.007	2.902	0.180
20%	50%	50%	20%	0.303	2.962	0.227	2.707	0.109	2.393	0.370	3.007	2.902	0.180
20%	50%	50%	50%	0.401	5.390	0.227	2.707	0.109	2.393	0.370	3.007	2.902	0.180
50%	20%	20%	20%	0.259	2.986	0.224	2.709	0.121	2.354	0.337	2.678	1.108	1.971
50%	20%	20%	50%	0.347	5.441	0.224	2.709	0.121	2.354	0.337	2.678	1.108	1.971
50%	20%	50%	20%	0.299	2.964	0.224	2.709	0.121	2.354	0.337	2.678	1.108	1.971
50%	20%	50%	50%	0.395	5.401	0.224	2.709	0.121	2.354	0.337	2.678	1.108	1.971
50%	50%	20%	20%	0.243	3.703	0.209	3.349	0.038	2.795	0.362	3.401	3.527	0.210
50%	50%	20%	50%	0.332	6.744	0.209	3.349	0.038	2.795	0.362	3.401	3.527	0.210
50%	50%	50%	20%	0.286	3.682	0.209	3.349	0.038	2.795	0.362	3.401	3.527	0.210
50%	50%	50%	50%	0.383	6.703	0.209	3.349	0.038	2.795	0.362	3.401	3.527	0.210

**True value**				**0.25**	**2.0**	**0.25**	**2.0**	**0.25**	**2.0**	**0.25**	**2.0**	**0.25**	**2.0**

Notes: GSD, geometric standard deviation. ERR, excess relative risk.

**Table 4.** Percentage bias in relative risk predicted at 0.1 Gy or 1 Gy with various sorts of dose error correction, using mean regression coefficients over *m*=500 dose+cancer simulations (as in Table 3)

Magnitude of error distribution (GSD)						Percentage bias in relative risk estimates by type of dose error correction							

Unshared Berkson error	Shared Berkson error	Unshared classical error	Shared classical error	Unadjusted		Regression calibration		Extended regression calibration		Monte Carlo maximum likelihood		Bayesian 2DMC with BMA	

				RR at 0.1 Gy	RR at 1 Gy	RR at 0.1 Gy	RR at 1 Gy	RR at 0.1 Gy	RR at 1 Gy	RR at 0.1 Gy	RR at 1 Gy	RR at 0.1 Gy	RR at 1 Gy

0%	0%	20%	20%	−0.01	7.67	−0.46	0.23	−0.46	0.23	−0.46	0.23	1.14	5.65
0%	0%	20%	50%	2.44	67.90	−0.46	0.23	−0.46	0.23	−0.46	0.23	1.14	5.65
0%	0%	50%	20%	0.30	8.16	−0.46	0.23	−0.46	0.23	−0.46	0.23	1.14	5.65
0%	0%	50%	50%	2.79	68.14	−0.46	0.23	−0.46	0.23	−0.46	0.23	1.14	5.65
20%	20%	20%	20%	0.16	13.51	−0.30	5.47	−1.07	0.24	0.57	7.55	5.82	9.61
20%	20%	20%	50%	2.77	78.78	−0.30	5.47	−1.07	0.24	0.57	7.55	5.82	9.61
20%	20%	50%	20%	0.48	14.04	−0.30	5.47	−1.07	0.24	0.57	7.55	5.82	9.61
20%	20%	50%	50%	3.13	79.07	−0.30	5.47	−1.07	0.24	0.57	7.55	5.82	9.61
20%	50%	20%	20%	1.06	30.64	0.46	21.06	−0.98	7.76	2.11	34.70	23.64	25.61
20%	50%	20%	50%	4.28	108.64	0.46	21.06	−0.98	7.76	2.11	34.70	23.64	25.61
20%	50%	50%	20%	1.42	31.23	0.46	21.06	−0.98	7.76	2.11	34.70	23.64	25.61
20%	50%	50%	50%	4.69	108.95	0.46	21.06	−0.98	7.76	2.11	34.70	23.64	25.61
50%	20%	20%	20%	1.02	30.59	0.43	21.02	−0.90	6.90	1.48	23.55	8.18	25.49
50%	20%	20%	50%	4.22	108.85	0.43	21.02	−0.90	6.90	1.48	23.55	8.18	25.49
50%	20%	50%	20%	1.40	31.17	0.43	21.02	−0.90	6.90	1.48	23.55	8.18	25.49
50%	20%	50%	50%	4.64	109.10	0.43	21.02	−0.90	6.90	1.48	23.55	8.18	25.49
50%	50%	20%	20%	1.56	52.18	0.90	40.26	−1.27	17.93	2.42	46.57	29.64	45.75
50%	50%	20%	50%	5.32	148.48	0.90	40.26	−1.27	17.93	2.42	46.57	29.64	45.75
50%	50%	50%	20%	1.95	52.84	0.90	40.26	−1.27	17.93	2.42	46.57	29.64	45.75
50%	50%	50%	50%	5.77	148.78	0.90	40.26	−1.27	17.93	2.42	46.57	29.64	45.75

Notes: GSD, geometric standard deviation.
